# Effects of berberine on pharmacokinetics of midazolam and rhodamine 123 in rats in vivo

**DOI:** 10.1186/s40064-016-2013-z

**Published:** 2016-03-29

**Authors:** Hua-Wen Xin, Xia Tang, Meng Ouyang, Jian-Xun Zhong, Wei-Liang Li

**Affiliations:** Department of Clinical Pharmacology, Wuhan General Hospital of Guangzhou Command, Wuhan, 430070 China

**Keywords:** Berberine hydrochloride, CYP3A, P-glycolprotein, Midazolam, Rhodamine 123, Pharmacokinetics

## Abstract

**Aim:**

To evaluate whether berberine hydrochloride (BBR) could modify the pharmacokinetic profiles of midazolam (MDZ), a substrate of CYP3A, and rhodamine 123 (Rh123), a substrate of P-glycolprotein (P-gp), in male rats.

**Methods:**

The rats were given with varied does of BBR or 75 mg/kg ketoconazole as a positive control for 10 days by intragastric administration. Single-pass duodenum perfusion of 20 mg/kg MDZ and inguinal artery canulated rats were used in the study. Plasma concentrations of MDZ and 1′-hydroxymidazolam (1′-OH-MDZ) were analyzed by high performance liquid chromatography (HPLC). The rats were given with varied does of BBR or 4 mg/kg verapamil as a positive control for 10 days by intragastric administration. Blood was obtained from the caudal vein of rats after single-pass intragastric administration of 5 mg/kg Rh123. HPLC was used to analyze the plasma concentrations of Rh123.

**Results:**

BBR produced similar results as the ketoconazole (positive control group) with a dose-dependent increase in the AUC_(0−t)_ and AUMC _(0−t)_ of midazolam except at the dose of 50 mg/kg (*p* < 0.01). And BBR could significantly increase the peak plasma concentrations (C_max_) of MDZ (*p* < 0.01), but reduce the clearance rate (CL_z_) and the apparent volume of the distribution (V_z_) of MDZ (*p* < 0.05). The results also indicated that BBR had no significant impact on the half-life period (t_1/2_) and the time to reach peak concentration (t_max_). Meanwhile, BBR could dose-dependently decrease AUC_(0−t)_ and AUMC_(0−t)_ of 1′-OH-MDZ significantly (*p* < 0.05), and expedite the clearance rate of 1′-OH-MDZ while gaining its apparent volume of distribution (*p* < 0.05), but had no significant impact on t_1/2_ and T_max_. The result also showed that BBR, except at the dose of 50 mg/kg, and the positive verapamil group could significantly increase the AUC_(0−t)_ and AUC_(0−∞)_ of Rh123 (*p* < 0.001), meanwhile raise C_max_ of Rh123 and shorten its V_z_ inversely (*p* < 0.05). Additionally, pre-treatment with BBR had no significant influence with the half-life period of Rh123, while significantly reduced its clearance rate (*p* < 0.05).

**Conclusion:**

The metabolism of MDZ and Rh123 was controlled by BBR. The results were most likely due to the inhibition by BBR on CYP3A enzymes and P-gp transporter.

## Background

Cytochrome P450 are important drug metabolism enzymes in vivo, involved in the metabolism of some important endogenous substances and more than 50 % of the clinical medicine. P-glycoprotein (P-gp)-mediated drug efflux and cytochrome p450 3A (CYP3A) metabolism within the P-glycoprotein (P-gp) mediats drug efflux and cytochrome P4503A (CYP3A) metabolism within the intestines creates a barrier that affects the absorption of many oral drugs (Benet et al. 1999; Zhou 2008). A number of studies have found that the change of the activity of P-gp will also cause the absorption and metabolism of P-gp substrates (Malati et al. 2012; Zhang et al. 2007). Therefore, a more in-depth understanding of the effects of drugs on the activity of CYP3A enzyme and P-gp will have an important significance on the evaluation of drug interaction.

In years of clinical practice, we had found that berberine hydrochloride (BBR) could significantly increase plasma concentration of cyclosporineA (CsA) in transplant recipients (Wu et al. 2005; Xin et al. 2006). Our studies showed that BBR could inhibit the CYP3A activity in vivo on rat and mouse liver, small intestine (Xin et al. 2004, 2005). We also confirmed that the pre-treated BBR combined with CsA or high doses of BBR alone could significantly alter mdr1a and mdr1b gene expression (Xin et al. 2002).

Based on the previous conclusion, the purpose of this experiment is to study the effects of BBR on CYP3A and P-gp in rats. Midazolam (MDZ), as an ideal CYP3A probe, has been widely used to evaluate the effects of drugs on CYP3A enzyme (Kim et al. 2002; Rogers et al. 2003; Xin et al. 2012). Rhodamine 123(Rh123) is the classic P-gp active substrate, and its elimination in vivo was only related with the activity of P-gp (Bao et al. 2012). Our laboratory had established detection methods for rat plasma midazolam, 1′-hydroxymidazolam (1′-OH-MDZ), and the blood serum concentration of Rh123 (Li et al. 2012). We studied the effects of BBR on MDZ and Rh123 metabolism in rats to evaluate the effects of BBR on CYP3A activity and P-gp transporter.

## Results and discussion

### Effect of berberine on midazolam and its metabolite pharmacokinetic experiments in rat

**Fig. 1:**
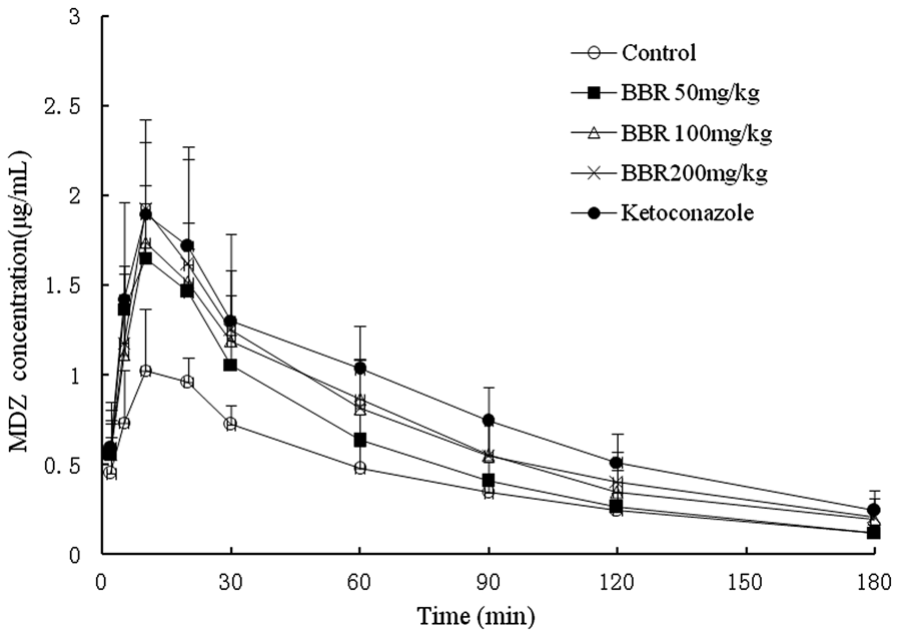
The effect of multi-dose of berberine or ketoconazole on the pharmacokinetics of midazolam in rats. *Data* present mean concentrations ± SD, n = 8

Figure 1 below presented the time-plasma concentration variation trend of MDZ after pre-treated with other drugs. As Fig. 1 showed, rat plasma concentrations of MDZ in oral BBR group and the positive ketoconazole group were higher than that in the negative group. Rat plasma concentrations of MDZ in the positive group (*p* < 0.05) were significantly higher than that in the negative group except at the point of 2 min. In addition, the rat plasma concentrations of MDZ were higher than that in the negative group. Therefore, BBR could dose-dependently increase the plasma concentration of MDZ.

**Fig. 2:**
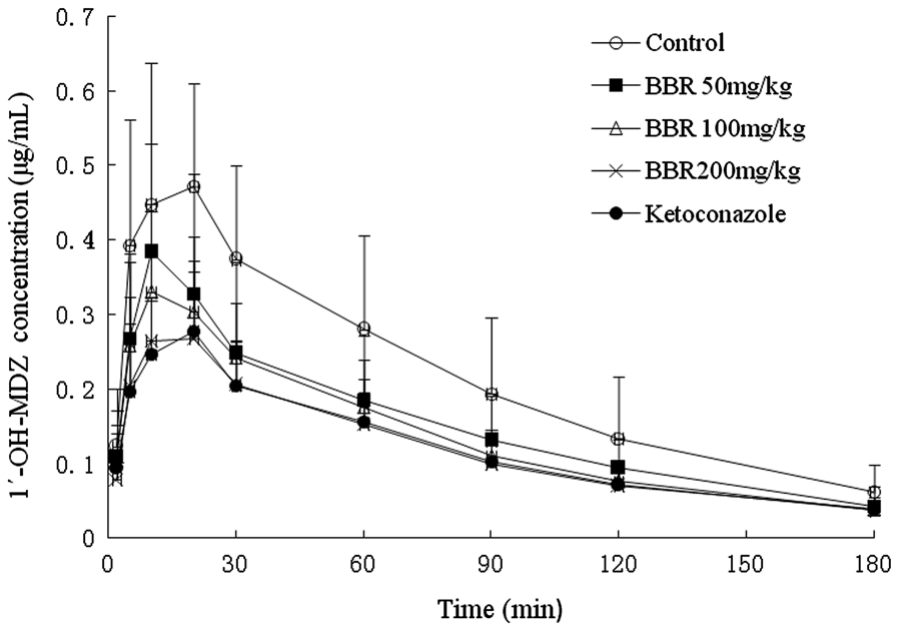
The effect of multi-dose of berberine or ketoconazole on the pharmacokinetics of 1′-hydroxymidazolam in rats. *Data* present mean concentrations ± SD, n = 8

Figure 2 showed the time-plasma concentration variation trend of 1′-OH-MDZ after pre-treating with BBR or ketoconazole. As Fig. 2 showed, BBR and ketoconazole could decrease rat plasma concentrations of 1′-OH-MDZ. It has been shown that ketoconazole and BBR (100 and 200 mg/kg) could significantly decrease rat plasma concentration of 1′-OH-MDZ except at the points of 2 and 10 min (*p* < 0.05). Moreover, data showed a significant reduction of rat plasma concentration of 1′-OH-MDZ after giving 50 mg/kg BBR between the point 20 to 90 min (*p* < 0.05).

Pre-treated with BBR or ketoconazole could alter some pharmacokinetic parameters of MDZ and 1′-OH-MDZ (Tables 1, 2). BBR at 200 and 100 mg/kg could decrease AUC_(0−t)_ and AUMC_(0−t)_of MDZ significantly (*p* < 0.01) in a dose-dependent way, while BBR of three dose groups could significantly increase the peak plasma concentrations (C_max_) of MDZ (*p* < 0.01). And all BBR groups could reduce the clearance rate (CL_z_) and apparent volume of distribution (V_z_) of MDZ (*p* < 0.05), while it had no significant impact on half-life period (t_1/2_) and the time to reach peak concentration (t_max_). AUC_(0−t)_, AUMC_(0−t)_ and C_max_ of MDZ in the 200 mg/kg group increased by 69.6, 67.7 and 87.6 % respectively, while the clearance rate and apparent volume of distribution of MDZ reduced by 37.3 and 36.4 % respectively.

**Table 1:** Dynamic parameters of midazolam after treated with BBR or ketoconazole in rats ($\overline{\text{x}}$ ± s, n = 8**)**

Parameters	Negative control group	BBR 50 mg/kg	BBR 100 mg/kg	BBR 200 mg/kg	Ketoconazole 75 mg/kg
AUC_(0−t)_ (μg/mL h)	1.25 ± 0.29	1.69 ± 0.31	2.03 ± 0.44***	2.12 ± 0.66***	2.47 ± 0.49***
AUMC_(0−t)_ (μg/mL h)	1.24 ± 0.37	1.49 ± 0.29	1.98 ± 0.56**	2.08 ± 0.75**	2.57 ± 0.65***
CL_z_ (L/h/kg)	14.89 ± 4.93	11.23 ± 1.8*	9.19 ± 2.31***	9.33 ± 4.09**	7.17 ± 1.85***
V_z_ (L/kg)	20.51 ± 3.63	12.58 ± 3.05***	12.12 ± 3.28***	13.05 ± 3.74***	10.85 ± 2.37***
t_1/2_ (min)	61 ± 18	47 ± 11	56 ± 14	61 ± 12	66 ± 23
t_max_ (min)	13 ± 5	12 ± 5	12 ± 6	11 ± 5	10 ± 3
C_max_ (μg/mL)	1.13 ± 0.25	1.69 ± 0.23**	1.84 ± 0.29***	2.12 ± 0.53**	2.21 ± 0.29***

Compared with the negative control group, * *p* < 0.05, ** *p* < 0.01, *** *p* < 0.001

**Table 2:** Dynamic parameters of 1’-hydroxymidazolam after treated with BBR or ketoconazole in rats ($\overline{\text{x}}$ ± s, n = 8)

Parameters	negative control group	BBR 50 mg/kg	BBR 100 mg/kg	BBR 200 mg/kg	ketoconazole 75 mg/kg
AUC_(0−t)_ (μg/mL h)	0.66 ± 0.28	0.46 ± 0.19*	0.42 ± 0.11**	0.36 ± 0.09***	0.37 ± 0.10***
AUMC_(0−t)_ (μg/mL h)	0.68 ± 0.34	0.47 ± 0.22*	0.41 ± 0.12**	0.37 ± 0.11**	0.37 ± 0.12**
CL_z_ (L/h/kg)	29.93 ± 11.89	44.9 ± 20.95*	45.17 ± 10.85*	50.99 ± 11.90**	50.66 ± 12.94***
V_z_ (L/kg)	41.61 ± 12.07	67.57 ± 32.43*	63.75 ± 18.66*	71.33 ± 26.01*	75.57 ± 23.57**
t_1/2_ (min)	61 ± 17	62 ± 8	58 ± 11	57 ± 12	61 ± 7
t_max_ (min)	17 ± 5	11 ± 4	12 ± 6	12 ± 7	13 ± 7
C_max _(μg/mL)	0.51 ± 0.16	0.44 ± 0.13	0.40 ± 0.08*	0.32 ± 0.07***	0.33 ± 0.09**

Compared with the negative control group, * *p* < 0.05, ** *p* < 0.01, *** *p* < 0.001

Meanwhile, BBR could decrease AUC_(0−t)_ and AUMC_(0−t)_ of 1′-OH-MDZ significantly (*p* < 0.05) in a dose-dependent way, and expedite the clearance rate of 1′-OH-MDZ while it gained apparent volume of distribution (*p* < 0.05), but had no significant impact on t_1/2_ and T_max_. AUC_(0−t)_, AUMC_(0−t)_ and C_max_ of 1′-OH-MDZ in the 200 mg/kg group reduced by 45.5, 45.6 and 37.3 % respectively while the clearance rate and apparent volume of distribution of 1′-OH-MDZ increased by 70.4 and 71.4 % respectively.

### Effect of berberine on the Rh123 pharmacokinetic experiments in rats

**Fig. 3:**
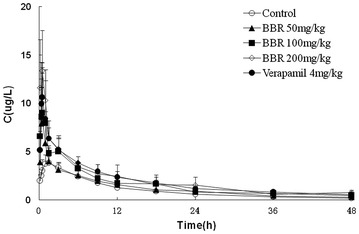
The effect of multi-dose of berberine or verapamil on the pharmacokinetics of rhodamine 123 in rats. *Data* present mean concentrations ± SD, n = 8

Pre-treated with different doses of BBR or verapamil raised the rat plasma concentration of Rh123 (Fig. 3). Pre-treated with BBR at 200 mg/kg or verapamil, the concentration was significantly higher than that in the negative group at the time point of 1 h and lasted to 48 h (*p* < 0.05). Furthermore, the increased concentration of Rh123 with high doses of BBR was similar to the increased concentration with positive drug verapamil at 20 min after gavage treatment. In conclusion, BBR could increase the plasma concentration of Rh123.

Table 3 showed the altered pharmacokinetic parameters of Rh123 after giving diverse drugs. In contrast to the negative group, 100, 200 mg/kg BBR and the positive verapamil group significantly increased the AUC_(0−t)_ and AUC_(0−∞)_ of Rh123 (*p* < 0.001), but reduced its clearance rate and apparent volume of distribution (*p* < 0.01). In addition, pre-treated with BBR, except at dose of 50 mg/kg, or verapamil could increase C_max_ of Rh123 (*p* < 0.05). Besides that, BBR had no significant influence with half-life period of Rh123, but the positive drug verapamil significantly prolonged the half-life period of Rh123 (*p* < 0.01). AUC_(0−t)_, AUC_(0−∞)_ and C_max_ of Rh123 in the 200 mg/kg group increased by 97.5, 101.7 and 268.5 % respectively, while the clearance rate and apparent volume of distribution of Rh123 reduced by 49.7 and 28.3 % respectively.

**Table 3:** Pharmacodynamic parameters of Rh123 after treated with berberine or verapamil in rats **(** $\overline{\text{x}}$ ± s, n = 8)

Parameters	Negative control group	BBR 50 mg/kg	BBR 100 mg/kg	BBR 200 mg/kg	Verapamil 4 mg/kg
AUC_(0-t) _(μg/L h)	48.36 ± 6.4	59.58 ± 13.37	77.51 ± 6.84***	95.49 ± 15.99***	93.01 ± 13.07***
AUC_(0−∞)_ (μg/L h)	50.97 ± 6.99	63.52 ± 14.52	84.42 ± 7.72***	102.8 ± 18.25***	105.8 ± 14.79***
CL_z_ (L/h/kg)	99.64 ± 14.01	81.95 ± 17.98*	59.63 ± 5.46***	50.11 ± 10.54***	47.96 ± 6.41***
V_z_ (L/kg)	1687 ± 219	1446 ± 347	1208 ± 293**	1209 ± 124***	1204 ± 205**
t_1/2_ (h)	11.78 ± 0.89	12.39 ± 2.9	13.97 ± 2.73	14.47 ± 1.88	17.41 ± 1.79**
t_max_ (h)	1.4 ± 0.96	0.4 ± 0.09	0.5 ± 0.29	0.27 ± 0.15	0.4 ± 0.09
C_max_ (μg/L)	4.41 ± 0.45	10.18 ± 5.59	11.78 ± 3.19*	16. 25 ± 8.65***	11.39 ± 2.76*

Compared with the negative control group, * *p* < 0.05, ** *p* < 0.01, *** *p* < 0.001

Cytochrome P450 3A (CYP3A) is the most abundant hepatic and intestinal phase I drug-metabolizing enzyme. It participates in the metabolism of approximately 50 % of drugs on the market and in the oxidation of endogenous and exogenous compounds. Many drugs are metabolized by CYP3A, an enzyme whose activity can affect many drugs’ effect and consequentially cause drug toxicity within patients. The enzyme’s activity can be induced or inhibited by many drugs, which would take part in a drug–drug interaction. P-gp is mainly distributed in the cell membrane and belongs to the ATP binding cassette (ABC) transporter superfamily member (Schwab et al. 2003). P-gp is found in various tissues such as the liver, brain, kidney and small intestine (Matheny et al. 2012). P-gp can reduce drug absorption in the intestine and increase drug elimination by the liver cell membrane.

CyclosporineA (CsA) is one of the most widely used immunosuppressants in organ transplant recipients. In years of clinical practice, we have found that BBR can significantly increase plasma concentration of CsA in transplant recipients. The combination of BBR and CsA can lower the necessary dosage of CsA to achieve a similar result. As a result, the combination can be an efficient means to economize the high cost of CsA (Wu et al. 2005). CYP3A and P-gp regulated the absorption and metabolism of CsA, which is the substrate of CYP3A and P-gp. Studies have shown that as a result of transplant recipients taking CsA, 56 % of the apparent oral clearance difference is induced by liver CYP3A4 enzyme activity and 17 % is induced by P-gp activity of the small intestine. Similarly, as a result of transplant recipients taking CsA, 32 % of the C_max_ difference was induced by liver CYP3A4 enzyme activity and 17 % is induced by P-gp activity of the small intestine (Lown et al. 1997). Therefore, CYP3A and P-gp play an important role in the ADME of CsA. We studied the influence of BBR on pharmacokinetics of MDZ and Rh123, which acts as the probes of CYP3A and P-gp respectively. The study explains the influence of BBR on the drug metabolism of CYP3A and P-gp in rats directly.

MDZ, a sedative-hypnotic drug, was mainly used for induction of anesthesia and preoperative administration. More and more researchers had used MDZ as a probe to study the function of CYP3A (Lee et al. 2002). MDZ was entirely metabolized by CYP3A but not the substrate of P-gp (Zhang et al. 2007). MDZ would be an ideal probe of CYP3A in a vivo or vitro study. MDZ is metabolized by CYP3A4 in the human body and by CYP3A1/2 in rats and mice. 1′-OH-MDZ was the main metabolite of MDZ (Chovan et al. 2007). Analyzing the variation of MDZ and 1′-OH-MDZ can evaluate the influence of drugs on CYP3A indirectly.

As a traditional Chinese medicine, BBR was widely used to treat diarrhea in clinical practice. Studies had shown that BBR had others pharmacology functions, such as anti-heart failure, anti-inflammatory, anti-platelet aggregation, lowering cholesterol and improving insulin resistance in recent years. We found that BBR could significantly increase the plasma concentration of CsA in renal transplantation patients, and could greatly improve the bioavailability of CsA without increasing the liver toxicity of CsA (Wu et al. 2005). In renal transplant recipients and healthy subjects, the pharmacokinetic study of CsA had also verified that the synergistic effect of BBR on the CsA (Xin et al. 2006). Since CsA was transported and metabolized by CYP3A and P-gp after oral absorption, we used MDZ as a CYP3A probe and Rh123 as a P-gp probe to study the effect of BBR on MDZ and Rh123.

In each BBR group, MDZ and 1′-OH-MDZ plasma concentration curves were between the negative control group and the positive control group. The plasma concentration of MDZ increased with the increasing doses of BBR, and the corresponding plasma concentration of 1′-OH-MDZ decreased with the increasing doses of BBR. In the high dose group of BBR, AUC_(0−t)_ and C_max_ of MDZ were significantly higher than that of the negative control group (*p* < 0.01). The clearance rate and the apparent volume of distribution decreased with increasing doses of BBR (*p* < 0.05). It showed that BBR could inhibit the MDZ clearance speed but it did not increase the rate of accumulation in the body. However, there was no significant difference in the half-life (t_1/2_) of MZD and T_max_. Accordingly, in each BBR group, the AUC_(0−t)_ of 1′-OH-MDZ was significantly lower than the negative control group (*p* < 0.05), the C_max_ in the high dose group was decreased, and the clearance rate and the apparent volume of distribution increased with the increasing doses of BBR (*p* < 0.05). BBR had no significant influence on t_1/2_ and t_max_.

In the low dose BBR groups, the Rh123 plasma concentration curves almost coincided with the negative control group. Low dosages of BBR had no significant increase of plasma concentration of Rh123. The Rh123 plasma concentration was increased gradually with increasing doses of BBR. 200 mg/kg BBR had almost equivalent effects with the positive control verapamil. This result showed that BBR dose-dependently inhibited the activity of P-gp. In the middle and high dose BBR groups, AUC_(0−t)_ of Rh123 was significantly higher than that in the negative control group (*p* < 0.01). C_max_ increased with the increasing doses of BBR. In the middle and high dose BBR groups, the clearance rate of Rh123 and the apparent volume of distribution (except for the low dose BBR group) decreased with the increasing doses of BBR (*p* < 0.05). This result also showed that BBR could inhibit Rh123 but not increase the accumulation in the body.

The above results showed that BBR inhibited the metabolism of MDZ and Rh123 in rats, and these effects were likely to be the inhibitory effect of BBR on CYP3A activity and P-gp. Our previous studies had shown that Schisandrin A was able to significantly inhibit MDZ metabolism in rats and that 8, 16, 32 mg/kg of Schisandrin A could make AUC_(0−t)_ of MDZ increase by 64.12, 86.88 %, 105.30 % respectively (Li et al. 2012). In this experiment, low, middle and high doses of BBR increased AUC_(0−t)_ of MDZ by 35.2, 62.4, 69.6 % respectively, and reduced AUC_(0−t)_ of 1′-OH-MDZ by 30.3, 36.4, and 45.5 % respectively. Low, middle and high doses of BBR increased AUC_(0−t)_ of Rh123 by 23.2, 47.9, 97.5 % respectively, and C_max_ was significantly increased. Thus, the inhibition of BBR on P-gp in low dose group is not strong. It was obvious that the inhibitory effect was dependent on the dosage of BBR. In addition, a study on the effect of BBR on P-gp in vivo had also reached the following results: using gavage with 100 mg/kg BBR for 2 weeks, the AUC_(0−t)_ of P-gp substrate digoxin increased by 69.18 % (Qiu et al. 2009), and this result was in strong agreement with our result of 100 mg/kg BBR 10 days after intragastric administration. However, the above study showed that after a 2-week pretreatment with BBR, the pharmacokinetic parameters of i.g. administered carbamazepine (CBZ, a substrate of CYP3A) and its metabolite carbamazepine 10,11-epoxide (ECBZ) were not significantly altered (Qiu et al. 2009). The reason for no significant changes in CYP3A activity by berberine using CBZ as a probe needs to be study further.

## Conclusions

Our data showed that the metabolism of MDZ (a probe of CYP3A) and Rh123 (a probe drug of P-gp) was controlled by BBR in rats. It could be due to the inhibition by BBR on CYP3A enzymes and P-gp transporter. Once further research confirms the inhibitory effect of BBR on P-gp or CYP3A4 in the human body, it would help to clarify the mechanism of enhancement of BBR on CsA in renal transplantation patients and thereby provide the exact evidence for the clinical application of BBR as a synergist of CsA.

## Methods

### Animals

#### Pharmacokinetic study

Healthy male Sprague–Dawley rats, weighing 250–280 g and 2–3 months of age, were purchased from Laboratory Animal Research Center of Wuhan University (Wuhan, China). The rats were housed in an accredited Laboratory Animal Center under controlled temperature (22 ± 2 °C) and a 12-h dark/light cycle. All animals were allowed to acclimate for a minimum of 3 days before the studies were initiated. The studies were designed in accordance with the protocols approved by the Wuhan General Hospital Institutional Review Board—Use and Care of Animal Committee and all experimental protocols involving animals were approved by the Ethics Committee of Wuhan General Hospital. The whole pharmacokinetic study was designed according to the Guidance for Industry Drug Interaction Studies from US FDA (2012) and the relevant guideline for pharmacokinetics and metabolic studies in human and animals from European Medicines Agency and Chinese FDA.

### Effects of berberine on midazolam and its metabolite

The rats were randomly divided into five groups with eight rats in each group. The rats in the treated groups were gavaged once daily with BBR at 50, 100 or 200 mg/kg for 10 consecutive days, and the rats were gavaged similarly with equal volumes of vehicle in the negative control group or ketoconazole (75 mg/kg) in the positive control group. On day 10, the rats were intraperitoneal injection with 2 % urethane dissolved. 30 min after the rats were treated with their respective drugs, midazolam was administered using single-pass duodenum perfusion at 20 mg/kg. Blood samples of about 0.6 ml were drawn from the femoral artery at 0, 2, 5, 10, 20, 30, 60, 90, 120 and 180 min after administration of midazolam. The rats were administrated with 3 ml of glucose saline (10 % glucose solution:physiological saline = 3:7) at 30 min. Blood samples were immediately heparinized and centrifuged at 3000 rpm/min for 10 min at 4 °C to obtain plasma samples, which were frozen at −40 °C until HPLC analysis.

### Effects of berberine on rhodamine 123

The rats were randomly divided into five groups with eight rats in each group. The rats in the treated groups were administrated in the same way for 10 consecutive days, and rats were gavaged similarly with equal volume of verapamil (4 mg/kg) in the positive control group. 30 min after the rats were treated with their respective drugs, Rh123 (5 mg/kg) was orally administrated. Blood samples were drawn from the caudal vein at 0, 5, 10, 20, 30, 60, 90 min and 3, 6, 9, 12, 18, 24, 36, 48 h after administration of Rho123, then the samples were immediately heparinized and plasma samples were obtained, which were frozen at −40 °C until HPLC analysis.

### Sample processing and HPLC analysis

The plasma sample (250 μl) from 1.3.1 was mixed with 20 μl of internal standard solution (diazepam) before the addition of 200 μl of buffer bicarbonate (pH 10) to alkalify the plasma drug and then 5 ml mixture (dichloromethane:hexane = 7:3, v/v) was mixed and shaken for 5 min. After vortex-mixing, the mixture was centrifuged at 3000×*g* for 10 min. After the upper aqueous phase had been removed, 4.5 ml of the organic phase was transferred to another tube and completely evaporated under nitrogen gas. The residue was dissolved in 250 μl of the mobile phase and 50 μl was injected into the HPLC system.

The HPLC analytical method for plasma 1′-hydroxymidazolam and MDZ was improved from an earlier report (Juřica et al. 2007), and it was described in our previous report (Li et al. 2012). A Hypersil BDS C18 column 4.6 mm × 250 mm equipped with a guard column, C_18_ BDS guard column 4.6 mm × 10 mm was kept at 40 °C. The mobile phase consisted of acetonitrile, methanol and 20 mM phosphate buffer (pH = 3.9) (20:42:38, v/v) and was pumped at a constant rate of 1.0 ml/min. The detection wavelength was set at 230 nm.

The HPLC analytical method for plasma Rh123 was described in our previous report (Xin et al. 2014). 150 μl of samples from 1.3.2 were mixed with 150 μl acetonitrile for 5 min to precipitate protein. After forming flocculent precipitate, 20 μl of supernatant was injected into the chromatographic column, a Hypersil BDS C18 column 4.6 × 150 mm, kept at 25 °C. The mobile phase consisted of acetonitrile and 20 mM phosphate buffer (pH = 4.0) (60:40, v/v) and was pumped at a constant rate of 1.0 ml/min. The fluorescence detection wavelength was set at 485 nm for excitation and 546 nm for emission.

### Statistical analysis

The estimation of pharmacokinetic parameters was conducted using DAS (Drug and Statistics) 2.0 analysis soft (Shanghai, China). One-way ANOVA followed by post hoc least significant difference (LSD) tests was used to determine group differences based on the assumption of equal variances for the pharmacokinetic parameters, and Tamhane’s T2 test was used in the case of unequal variances (SPSS 13.0). A *p* < 0.05 was considered statistically significant.

## Abbreviations

BBR: berberine hydrochloride
CsA: cyclosporineA
MDZ: midazolam
Rh123: rhodamine 123
1′-OH-MDZ: 1’-hydroxymidazolam
P-gp: P-glycoprotein
CYP: cytochrome P450
CYP3A: cytochrome P450 3A
C_max_: peak plasma concentration
t_max_: the time to reach peak concentration
t_1/2_: half-life
AUC_(0−t)_: area under the plasma concentration–time curve
AUMC_(0−t)_: area under the first moment curve
CL_z_: apparent clearance
V_z_: apparent volume of distribution
HPLC: high performance liquid chromatography
